# Relational Humor and Identity Framing in the “Virgin vs. Chad” Meme Format

**DOI:** 10.3390/bs15091251

**Published:** 2025-09-14

**Authors:** Ana Yara Postigo-Fuentes

**Affiliations:** Institut für Romanistik, Heinrich-Heine Universität, Universitätstraße 1, 40225 Düsseldorf, Germany; ana.postigo.fuentes@hhu.de

**Keywords:** extremist narratives, far-right digital cultures political memes, multimodal discourse analysis, humour and ideology, identity and belonging

## Abstract

Extremist narratives combine two relational dynamics: the in-group is portrayed as both socially superior and simultaneously victimized by an antagonistic out-group, which legitimizes hostility or defensive solutions. Despite their relevance, such narratives remain comparatively understudied. To date, little research has examined how extremist narratives are represented through memes, and particularly how humour operates within memetic forms. This article develops and tests a three-layered analytical framework for examining humour in extremist digital cultures. The framework integrates insights from narrative studies, multimodal discourse analysis, and humour theory to capture how memes condense antagonisms, stabilize symbolic contrasts, and calibrate affective positioning. The *Virgin* vs. *Chad* meme format is used as a case study due to its binary archetypal structure and recurrent circulation in Spanish far-right meme ecologies. The study draws on 1225 posts on X (May–August 2024), from which 17 memes employing the format were selected for in-depth qualitative analysis. The findings show that the format performs symbolic compression by staging binary oppositions between in-group and out-group identities, typically valorizing figures associated with nationalism, masculinity, and epistemic certainty while delegitimizing those linked to progressivism, pluralism, or emotional expressiveness. These meanings are stabilized through repeated visual and typographic conventions, including character archetypes, split-panel layouts, and indexical stylization. Humour arises through devices such as irony, reversal, exaggeration, and incongruity, which render these oppositions as recognizable contrasts. By integrating insights from humour theory, narrative framing, and multimodal discourse analysis, the article contributes a methodological model for examining how memes condense and circulate antagonistic distinctions in online political ecologies.

## 1. Introduction

In digital environments, humour takes on new affordances linked to multimodality and rapid circulation (Attardo, 2024a). It contributes to relational practices by reinforcing group belonging, modulating proximity between users, and enabling the indirect expression of beliefs through irony and ambiguity (Attardo, 2024b; Dynel, 2020). In online subcultures, meme-based humour operates as a communicative strategy that aligns affect and marks group identity through contrast with perceived outsiders (Das, 2023; McSwiney et al., 2021).

While humour can serve as a vehicle for spreading different political ideas, there has recently been a particular focus on far-right digital spaces concealing them (Aladro Vico & Requeijo Rey, 2023; Fielitz & Ahmed, 2021). In these environments, humour stylizes ideological content in resonant and simplified forms, broadening its reach while deflecting critique (De Keulenaar, 2023; Schmid et al., 2024; Topinka, 2018). It thus acts simultaneously as signal and shield—expressing affiliation while managing reputational risk (Postigo-Fuentes, 2025a).

Regarding memes, the concept was first introduced by Dawkins (1976) as a unit of cultural transmission, analogous to a gene, that propagates through processes of imitation and variation. In digital contexts, memes have come to signify multimodal artefacts—typically combinations of text and image—that circulate rapidly and invite endless recontextualisation (Shifman, 2014; Osterroth, 2020). While they are often humorous, humour is not intrinsic to memes; rather, their defining property lies in their adaptability and *collective semiosis* (Osterroth, 2015), as meaning is constantly reshaped through iterative participation (Nowotny & Reidy, 2022). What distinguishes memes from other forms of subcultural discourse is precisely this modularity and virality, which allow them to condense complex ideas into symbolic shorthand and circulate them widely.

From a discourse-theoretical perspective, memes are not neutral carriers of content but vehicles of power: they structure visibility and legitimacy (Foucault, 1972) and act as nodal points where antagonisms are fixed within ideological struggles (Laclau, 2005). In extremist digital cultures, this capacity becomes particularly salient, as memes frame oppositional identities and moral hierarchies through stylized contrasts (Sun et al., 2025; Zannettou et al., 2018). Also, their humour often depends on subcultural literacies that reinforce insider status and gatekeep belonging, enabling inclusion, enforcing exclusion, and reframing ideological discourse through ironic detachment (Attardo, 2024a; McSwiney et al., 2021).

While research on far-right online cultures has increasingly examined memes as vehicles of ideological messaging (e.g., Fielitz & Ahmed, 2021; Topinka, 2018; Zannettou et al., 2018), the role of humour in these contexts has rarely been approached through the lens of interpersonal communication. Extremist narratives are not only political but also relational: they define who belongs and who is excluded, managing identity gaps, regulating disclosure, and disciplining interaction through ridicule and irony (Postigo-Fuentes et al., 2024). These dynamics are deeply affective. As Ahmed (2014) notes, emotions can “stick” to figures and circulate through discourse, shaping how groups come to feel aligned or opposed. Recent research on radical right populism similarly shows that narratives mobilize fear, resentment, and pride as central resources for identity construction (Gianolla et al., 2024).

In meme ecologies, Chad and Virgin function not only as humorous archetypes but also as affective anchors, invested with pride, authority, shame, or derision depending on their narrative positioning. This study addresses that gap by showing how the “Virgin vs. Chad” meme condenses extremist narrative logics into humorous contrasts between in- and out-groups. By analyzing this format, we show how humour operates as a mode of argumentation that simultaneously legitimizes exclusionary worldviews and structures interpersonal alignment in digital environments.

**Figure 1:**
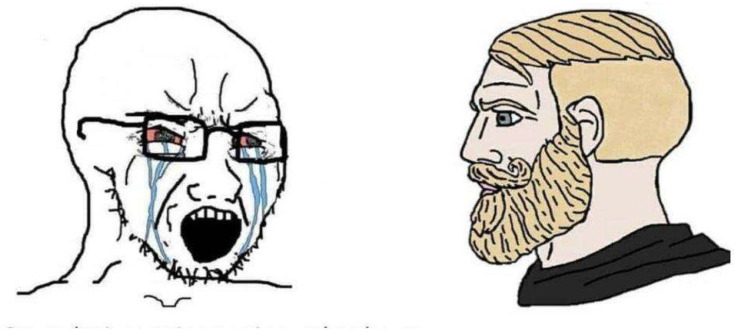
“Virgin vs. Chad”. Self-created image using an automatic meme generator.

Initially conceived as satire of masculinity and social performance, the “Virgin vs. Chad” binary has circulated across diverse online contexts. While not exclusive to far-right discourse, it has been repeatedly appropriated as a vehicle for contrastive archetypes that express normative and moral judgments. This study focuses on its function within far-right meme ecologies, where Virgin and Chad do not merely appear as humorous figures, but as symbolic positions that encode hierarchies of value and ideological stance (Postigo-Fuentes, 2024; Sun et al., 2025). Figure 1 shows the most common way of representing Chad vs. Virgin today.

Using Spanish-language meme ecologies as empirical context, the article examines how archetypal figures like Virgin and Chad acquire and convey ideological, affective, and identity-related significance within meme cultures. The analysis approaches the “Virgin vs. Chad” meme as both a narrative structure and a relational index.

To do so, this article develops and tests a three-layered framework for analyzing humour in extremist narratives as they circulate through memes. The aim is not to provide exhaustive coverage of meme ecologies, but to demonstrate how this model can account for the narrative, semiotic, and humorous dimensions of extremist narratives. The Virgin vs. Chad meme is selected as a case study because its binary archetypal structure makes it particularly apt for examining how ideological antagonisms are staged, compressed, and rendered humorous in far-right Spanish meme cultures. Accordingly, the three layers create a prism from which to understand the construction of identity and the relationship between in-group and out-group through the “Virgin vs. Chad” meme format.

### 1.1. Humour and Narrative as Relational Structures

Extremist narratives are not transmitted exclusively through explicit ideological claims or actors. Instead, they often emerge through the repeated framing of antagonistic relations between morally charged insider and outsider groups (Berger, 2018; Glazzard, 2017). Such narratives can therefore emerge in any discourse. Their appearance in different types of discourses at the same time can lead to their legitimisation. Through the processes of *memiosis* and viralisation (Attardo, 2024a; Osterroth, 2019), memes are particularly suited to this process. They also rely on symbolic compression, repetition, and affective cues to represent opposition in stylized, easily recognizable terms. Chang et al. (2024) highlight *generative memesis*, the tendency for stylistic repetition itself to become a meaning-making force. Viralisation, memeiosis, and generative memesis together explain how certain visual and affective patterns become recognizable and socially salient across contexts, often acquiring stability that transcends individual authorship.

Extremist narratives tend to follow a recurring structure (Postigo-Fuentes et al., 2024): they define an in-group presented as morally legitimate—often portrayed as vulnerable or under threat—and contrast it with an out-group positioned as dangerous or deficient. This binary is typically accompanied by a diagnosis of the out-group’s harmful influence, a stance toward that perceived harm, and a proposed response, which may range from distancing and exclusion to more aggressive forms of resolution.

Humour can contribute directly to the operation of this structure. It enables users to signal stance and affiliation without overt political expression. Through shared cues and evaluative contrast—what is laughed at, who is mocked, and how recognition is managed—humour shapes alignment. In the context of extremist narratives, it functions as an indexical resource for reinforcing group boundaries and authorizing exclusion, particularly when mediated through irony or exaggeration (McSwiney & Sengul, 2024; Topinka, 2018). Humour in digital culture is polyvalent and may also operate in ways that are playful or community-building (Topinka, 2018). It also can offer a mode of communication that is affectively charged and socially consequential, even when framed as playful or unserious (Mihăilescu, 2024).

As vernacular sources document (Know Your Meme, 2017), the “Virgin vs. Chad” format reflects these dynamics. It stages two figures—Virgin and Chad—as contrasting embodiments of conduct, disposition, and social value. Virgin is often rendered as anxious, verbose, and deferential; Chad as composed, affectively restrained, and assertive. These portrayals do not only signify personality types—they function as compressed moral positions. Chad, enregistered through internet slang and meme culture as an archetype of confident dominance, increasingly accrues ideological associations in far-right discourse, where he may represent ethnonationalist or misogynist stances (Monti, 2025). Virgin, often overlapping with Wojak variants, becomes his foil, associated with weakness, excess, or pluralist positions. The binary’s stability—but also its adaptability—make the format a productive case for analyzing how extremist narratives condense ideological antagonisms into humorous contrasts.

This structure justifies the first layer of analysis in the article. Understanding the ideological work of the meme requires attention to how it constructs oppositional roles, frames legitimacy, and produces alignment within an extremist narrative. Before turning to questions of form or affect, it is necessary to examine how narrative contrast shapes the moral and relational dimensions of the meme’s meaning.

### 1.2. Meme Grammar and the Semiotic Constraints of Archetypes

Memes are the most frequently tracked narrative units in political communication research in social media (Schmid et al., 2024; Sun et al., 2025; Zannettou et al., 2018), due to their high visibility and circulation via remix, aesthetic transformation, and recontextualization. Memes function as multimodal texts shaped by participatory remix (Milner, 2016; Shifman, 2013). Their recognizability relies on compositional conventions—such as visual grammar (Kress & van Leeuwen, 2001) and intertextual reference—that organize meaning across visual and textual elements. In this context, the “Virgin vs. Chad” format establishes contrastive relations through recurring features: posture, gaze, spatial positioning, and typographic voice. These cues invite attitudinal interpretation and guide evaluations of behaviour, affect, and social positioning. Rather than viewing it in isolation, the format is best understood as one semiotic node within broader ideological circuits, where online humour interacts with platform logics, media ecologies, and offline debates. “Chad” and “Virgin” function as condensed archetypes composed of visual, gestural, and textual features. The term *Chad* did not originate within far-right discourse. In the 1990s it circulated as a pejorative label for stereotypical “alpha males,” often popular athletes perceived as physically dominant but intellectually shallow. By the mid-2000s, the figure had entered online slang, later crystallizing in memes such as *Chad Thundercock* and *Gigachad*, which exaggerated his physique and stoic demeanour. From these beginnings, Chad became enregistered as an archetype of bold confidence and indifference to social sanction (Agha, 2024).

The “Virgin vs. Chad” meme, emerging around 2017, humorously contrasted this archetype with the socially awkward “Virgin,” establishing the contrastive template analyzed here. The *Virgin* figure draws on a longer genealogy of pejorative portrayals of male inadequacy and social awkwardness. While not attached to a single stable visual form, it resonates with incel discourses of sexual failure, the “beta male” stereotype, and broader tropes of maladjusted youth. Online forums such as 4chan popularized the label *Virgin* in the 2010s as shorthand for insecurity, passivity, and exclusion from desirability. Unlike *Chad*, which consolidated around increasingly iconic visual forms, *Virgin* remained diffuse, taking shape mainly through exaggerated bodily cues—slouched posture, averted gaze, hesitant gait—that came to symbolize weakness and deviance.

This figure often merges with “Wojak” (or “Feels Guy”), a minimalist line-drawn character widely used as a template to depict emotional instability, insecurity, or ideological excess (Figure 1). Wojak variants—including “soyjak” or “NPC Wojak”—frequently serve as the Virgin’s visual counterpart, foregrounding traits of verbosity, affective excess, or political naïveté. This porous overlap further stabilizes the contrastive opposition between the laconic, stoic Chad and the over-expressive Virgin/Wojak.

The processes of enregisterment and semiotisation are sustained through viralisation and, above all, memeiosis (Attardo, 2024a; Osterroth, 2019). Viralisation, through near-identical forwarding, reinforces the recognizability of the figures, while memeiosis—repetition with modification—allows for contextual adaptation. Across iterations, some features are consistently retained and thus “frozen,” giving *Chad* a relatively stable indexical meaning, while others are reconfigured or discarded. In this sense, *Chad* functions as a multimodal signifier: a recognizable figure whose core associations persist across settings, but which can also acquire additional meanings through contextual variation.

Precisely this adaptation to the variation of the context implies that there is a process of semiotisation (Kress & van Leeuwen, 2001), i.e., the acquisition of new meanings. In Spanish-language far-right meme ecologies, Chad is often framed as the *Verdadero Español*—the True Spaniard—positioned against *la Anti-España* (Forti, 2024). This frequently used structure in Spanish extremist narratives draws on historical binaries, reframed through meme aesthetics. Chad is often aligned with ethnonationalist identity and masculinist pride, while Virgin is linked to cosmopolitanism, technocracy, or political naiveté.

Although adaptable, these figures are constrained in how they signify. Chad may index confidence, assertiveness, or affiliation with nationalist or traditionalist positions, but not traits such as hesitation or emotional openness. These constraints serve a communicative function: they stabilize the figure as a marker of certainty and coherence (Postigo-Fuentes, 2025b). Virgin, by contrast, is characterized through visual and textual cues associated with insecurity, excess, or ideological deviation. These associations vary but often align with progressive or pluralist figures, depending on the interpretive setting.

The circulation of these archetypes is shaped not only by repetition but also by the affordances and tendencies of platform infrastructures. While the workings of recommender algorithms remain opaque and uneven across contexts, scholars have noted that platforms such as TikTok or Instagram tend to favour stylistic coherence and emotionally charged content, embedding what Zulli and Zulli (2022) term “memetic logic” into their recommendation systems. In this sense, the prominence of certain visual and affective patterns reflects not only repetition but also the generative force of memetic circulation (Chang et al., 2024), through which style itself acquires semiotic weight.

This justifies the second analytical layer of the study: the semiotic and linguistic grammar of the meme. By attending to how archetypes are constructed, constrained, and circulated through formal features and platform affordances, it becomes possible to analyze how contrastive positions are made interpretable, repeatable, and available for ideological uptake.

### 1.3. Humour as Ideological Calibration in Meme Discourse

Humour functions as a mechanism for encoding stance and regulating interpretive boundaries. It enables users to express evaluative positions without direct assertion, often relying on contrast, exaggeration, or irony to signal alignment (Godioli et al., 2025). Within these conditions, humour contributes to how alignment is signalled and received. Schmid et al. (2024) observe that memes combining humour with ideological framing tend to achieve broader circulation. Similar dynamics are highlighted by Fielitz and Ahmed (2021) and Schwarzenegger and Wagner (2018), who show how humorous antagonisms amplify resonance and diffuse more effectively in far-right online cultures. This aligns with broader accounts of meme spreadability (Shifman, 2013; Milner, 2016) and networked diffusion (Zannettou et al., 2018). In the context of extremist narratives, these functions are particularly relevant. Humour facilitates engagement while reducing interpretive commitment, making it a strategic resource for positioning content within contested discursive spaces (Godioli et al., 2025). Also, it reduces the perceived seriousness of a message while maintaining its evaluative force, facilitating circulation and lowering reputational risk. This makes it a key resource for establishing group cohesion, delegitimizing outsiders, and managing ambiguity in politically sensitive content (Fielitz & Ahmed, 2021; Schwarzenegger & Wagner, 2018).

To account for these dynamics, the third analytical layer draws on the General Theory of Verbal Humor (GTVH) (Attardo, 2024a), adapted for multimodal analysis. The GTVH proposes that humour operates through six interrelated dimensions: script opposition, logical mechanism, situation, target, narrative strategy, and language. In the “Virgin vs. Chad” format, oppositions are established through the spatial and visual organization of characters. Script oppositions—such as strength versus weakness or detachment versus emotionality—are visually encoded in posture, expression, and design. The logical mechanism often involves contrast or incongruity between figures, while the situation is represented as a stylized or archetypal interaction. These compressed scenarios rely on cultural fluency for interpretation and invite rapid stance recognition.

Targets are marked through visual and textual cues that index difference, often by exaggerating certain traits. This targeting plays a central role in delineating in-group and out-group positions, which is consistent with the boundary-defining logic of extremist discourse. Narrative strategy is typically minimal, often involving a two-part structure that frames one figure as initiating speech or action and the other as embodying resolution. Language is rendered through typography and tone, which function indexically: Chad’s text is brief and assertive; Virgin’s is often dense or stylised to evoke disalignment.

In the Virgin vs. Chad format, these elements how the meme is read, by whom, and under what affective conditions. Humour works here to stabilize the archetypes, to frame the opposition between them, and to render ideological stances affectively appealing. Crucially, this process relies on the meme’s multimodal affordances—visual compression, typographic contrast, and iterative variation—rather than on propositional argumentation alone.

## 2. Materials and Methods

### 2.1. Data Collection

This study draws on a curated subset of memes collected as part of the European Horizon ARENAS project. The dataset comprises 1225 Spanish-language X Posts published between 15 May and 5 August 2024. We originally selected this period because it encompassed two events expected to be productive for antagonistic discourse: the European Parliament elections and the Paris Olympic Games. Both were anticipated to trigger ideological contestation and therefore to surface in memetic antagonisms. While these events were not explicitly thematized in the “Virgin vs. Chad” instances found in our dataset, the meme nonetheless appeared recurrently in this period, condensed around broader ideological binaries rather than topical references. Posts were manually coded into four categories: “humour,” “potential memes,” “potential Chads/Virgins,” and “none.” From this corpus, seventeen memes that explicitly employed the “Virgin vs. Chad” template were selected for close analysis.

The selected dataset does not aim to represent the entirety of far-right meme discourse, but instead enables in-depth, interpretive analysis of a format that has become symbolically dense and ideologically saturated within Spanish meme ecologies, which makes them analytically significant despite their small number (Danino, 2018). They condense an archetypal opposition central to far-right identity work and recur widely in other corpora of extremist digital discourse. The restricted size of the sample is justified by the qualitative and interpretive nature of the study: the aim is not statistical generalization but to trace recurring semiotic and narrative processes in sufficient depth to test and refine the proposed analytical framework. Meanings are treated as context-dependent and culturally mediated, shaped by shared literacies, subcultural references, and ideological cues circulating within the communities that produce and engage with these memes. Rather than a limitation, this situatedness is understood as integral to examining the indexical and relational dynamics of meme-based ideological expression. Future research could extend this analysis to larger corpora to examine the broader applicability of these patterns.

### 2.2. Analytical Framework

This study applies a three-layered analytical model to examine how political antagonisms are constructed, visualized, and affectively encoded through the “Virgin vs. Chad” meme format. While the framework is applicable to other meme templates, we focus here on Virgin vs. Chad because its binary staging of archetypal figures offers a productive lens for analyzing identity representation and in-group/out-group dynamics in far-right digital discourse.

The first layer draws on research on extremist narratives and antagonism to show how the format organizes oppositional roles and moral evaluations. We built our own framework (Postigo-Fuentes et al., in press), building on the ones of Glazzard (2017) and Ingram (2016), as well as discourse-historical work (Wodak, 2021). It operationalizes recurrent components of extremist narratives (Postigo-Fuentes et al., 2024)—in-group construction, out-group construction, problematizations, variations, ideological perspective, implied solutions. In the Virgin vs. Chad template, these components are compressed into archetypal figures and short textual cues, allowing the meme to stage conflict and prescribe stance rapidly. This layer serves our objective by identifying which antagonisms are being told and what resolutions are being implied—whether exclusion, ridicule, or symbolic affirmation.

In the second layer, we adapt multimodal discourse analysis (Kress & van Leeuwen, 2001; Machin & Mayr, 2012) and social-semiotic insights into enregisterment (Agha, 2024), supplemented by meme-studies accounts of digital vernaculars (Shifman, 2013; Milner, 2016). We distinguish (a) intra-memetic affordances—character types, layout (e.g., split panels), speech/text styles, visual shorthand (e.g., jawline, tears, rainbow hair), and template grammar—and (b) contextual-discursive affordances—ideological literacy, meme literacy, intertextual anchors, speech enregisterment, and normative gatekeeping. Together these capture how recognizable visual–textual cues stabilize the reading of Chad/Virgin as evaluative types and how audience-specific literacies make the antagonism instantly readable. This layer serves our objective by specifying which semiotic resources cue value-laden interpretations and by showing how archetypes become legible as moral positions.

In the third layer, rather than treating humour as incidental, we follow the General Theory of Verbal Humour (GTVH) to analyze script oppositions, logical mechanisms (irony, reversal, exaggeration), situation, target, narrative strategy, and language (Attardo, 2024a, 2024b; Vandaele, 2010), complemented by work on far-right humour as boundary-work (Fielitz & Ahmed, 2021; McSwiney & Sengul, 2024). In our model, this layer is diagnostic: it asks what background information and affective positioning are presupposed to “get the joke,” and how those presuppositions channel identification with the in-group and ridicule of the out-group. This helps us avoid purely moral judgments and instead reconstruct the interpretive conditions under which a meme’s humour functions. This layer serves our objective by showing how affect is organized to secure stance and belonging. Taken together, the three layers offer a portable, comparative tool for meme analysis: Layer 1 identifies the antagonistic story being told; Layer 2 explains how that story is made visually and linguistically legible; Layer 3 shows how humour calibrates affect and alignment. Although tailored here to Virgin vs. Chad, the model can be potentially extended to other meme formats that combine archetypal opposition, semiotic compression, and humorous framing.

## 3. Results

### 3.1. Narrative Structural Layer

Across the dataset, memes consistently deploy binary oppositions to construct symbolic hierarchies, diagnose sociopolitical problems, and imply ideological resolutions. These oppositions function as affectively charged scripts, where “Chad” is positioned as the paradigmatic in-group figure—hypermasculine, nationalist, stoic, and often essentialized through cultural or biological determinism. By contrast, “Virgin” and related out-group characters are feminized, racialized, emotional, or conspiratorial. In this context, the Virgin figure is frequently associated with liberal, leftist, globalist, or progressive agendas. A summary of how these structures show up in the data can be found in Table 1.

These memes construct a worldview in which modernity is framed as decline, inversion, or disruption. The present is depicted through signs of weakness, confusion, and betrayal, while resolution is suggested through archetypes associated with masculine strength, national renewal, or spiritual discipline. Whether articulated through nostalgic imagery (e.g., Crusader Chad) or reframed in ascetic contemporary terms (e.g., Monk Chad), the structure remains consistent: confrontation is valorised over negotiation, and ideological purity is framed as a moral imperative. In-group construction is achieved through selective incorporation of historical, cultural, and political references. Chad may appear in different forms, such a Christian knight or a nationalist figure. Across these variants, he is consistently characterized by affective restraint, physical control, and unwavering conviction. These traits are not incidental but serve as semiotic cues of sovereign identity—opposed to emotional complexity or interpretive ambiguity.

By contrast, out-group figures are marked by excess, instability, or perceived moral deficiency. Feminists, LGBTQ+ individuals, immigrants, Muslims, and progressives are typically portrayed as verbose, socially over-integrated, or emotionally unregulated. In some cases, caricatured Jewish figures are used to suggest conspiratorial influence, drawing on established antisemitic tropes that frame cultural change as covert manipulation. Even centrists or moderate conservatives may be included among those portrayed as ideologically unreliable or insufficiently committed.

The meme format foregrounds several recurring frames of problematization: cultural decline linked to feminism or progressive values; demographic anxiety tied to immigration and birth rates; perceived betrayal by national elites, often in relation to Israel, the European Union, or multiculturalism; and the erosion of Christian or traditionalist identities. These concerns are presented not as topics for deliberation but as moral threats that require assertive responses.

**Figure 2:**
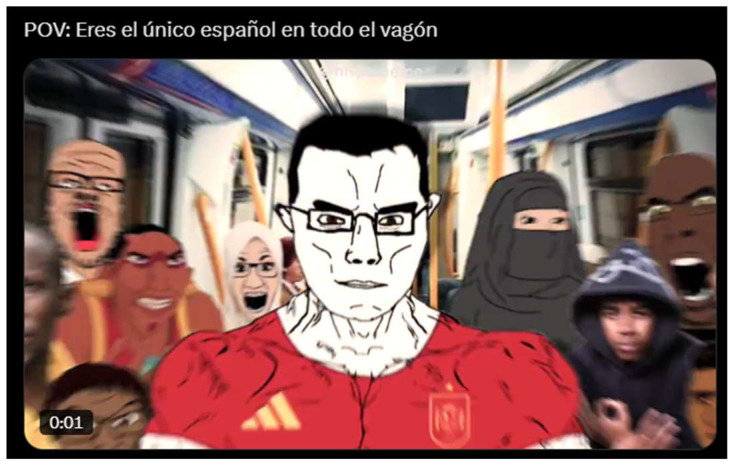
True Spaniard in train. Caption: “POV: You are the only Spaniard in the whole Wagon”.

Figure 2 shows an example of this narrative logic appears in a meme portraying Chad in a Spanish national football jersey, surrounded by caricatured immigrants and Muslims on a subway carriage. The in-group is symbolized through Chad’s embodiment of national identity, marked by restraint and centrality, while the out-group is constructed through racialized and religious Others, depicted as overwhelming his environment. The problematization is thus dramatized as demographic anxiety and cultural displacement: the “true Spaniard” becomes a lone figure in a hostile or alien context, where national identity is represented as besieged and in need of defence.

Solutions, when suggested, tend to emphasize the restoration of patriarchal authority, national unity, and ideological coherence. The format does not accommodate irony, pluralism, or ambiguity. Instead, it privileges assertiveness and moral clarity. The meme’s binary structure itself contributes to its ideological function: by reducing complex antagonisms to recognizable visual and narrative contrasts, it reproduces a simplified framework for understanding identity, conflict, and legitimacy.

**Figure 3:**
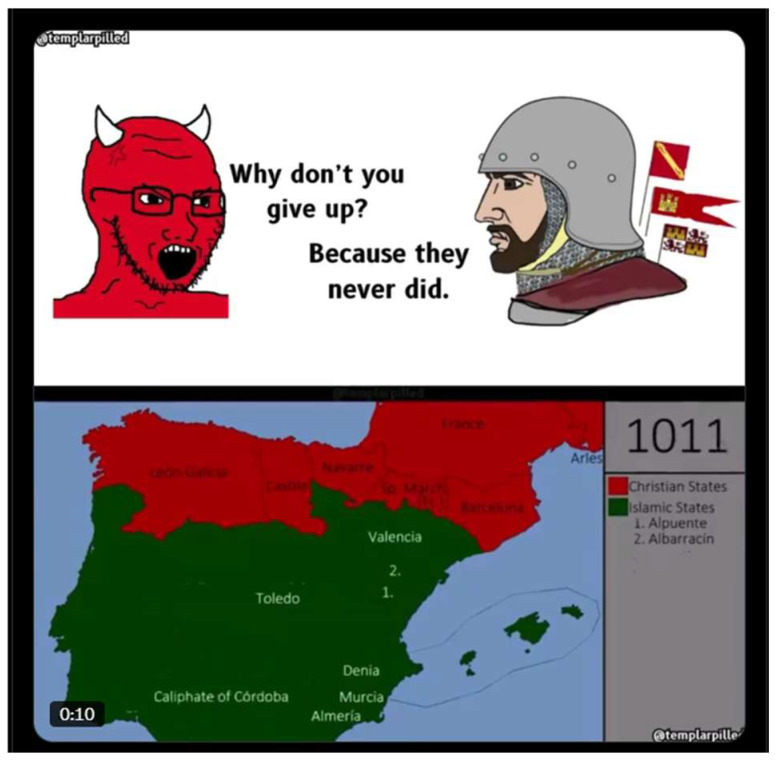
Reconquista Chad vs. Devil Virgin. The legend of the graph shows “Christian states” in red and “Islamic states” in green.

A meme that explicitly stages this logic shows a Crusader figure facing a devil-like Wojak who urges surrender, to which the knight replies, “Because they never did,” followed by a map of the Reconquista (Figure 3). Here, the in-group is constructed through a nostalgic invocation of medieval Christian warriors, whose perseverance against Islamic rule is reframed as a timeless model of civilizational fortitude. The out-group is symbolized through the devil caricature and the historical presence of Muslims, re-signified as an enduring threat. The problematization lies in the implicit analogy between past and present: just as Christian Spain once faced an existential struggle, contemporary society is portrayed as similarly endangered by cultural erosion and demographic change. The implied solution is neither negotiation nor pluralism but confrontation, framed as fidelity to a heroic lineage. This structure exemplifies how the meme format collapses modern anxieties into historical templates, positioning purity, strength, and perseverance as the only legitimate responses to decline.

### 3.2. Aesthetic Layer

This second analytical layer examines the semiotic and multimodal linguistic structuring of ideological content within the “Virgin vs. Chad” meme format. It distinguishes between intra-memetic affordances (Table 2)—those embedded within the meme’s compositional design—and contextual-discursive affordances (Table 3), which rely on audience familiarity with ideological, cultural, or memetic frameworks. Together, these affordances allow the meme to function as a condensed visual ideology: a form that trains recognition, elicits stance, and regulates discursive inclusion.

These compositional conventions reinforce binary logics through spatial, gestural, and textual contrast. Chad typically occupies the narrative or visual endpoint (e.g., bottom-right panel), anchoring the meme’s ideological resolution. His features—defined jawline, upright posture, direct gaze—function as shorthand for embodied sovereignty. His speech is sparse and aphoristic, indexing emotional restraint and epistemic authority. Utterances such as “No.” or “Hmm.” are not empty; they signify moral detachment and ideological clarity.

In contrast, Virgin and NPC figures are rendered as over-verbalized and affectively unstable. Their speech is dense, slogan-laden, or emotionally exaggerated. These characters frequently cite familiar progressive slogans not as expressions of conviction, but as signs of ideological vacuity. Such utterances operate as enregistered speech styles, recognizable to in-group audiences as markers of incoherence or naïveté.

Formally, the memes are designed for rapid legibility. Visual conventions—colour-coded hair, symbolic accessories, exaggerated gestures—encode ideological positions with minimal textual effort. This semiotic compression allows for quick interpretation, high reproducibility, and portability across platforms. The meme’s visual economy is instrumental to its discursive efficiency.

**Figure 4:**
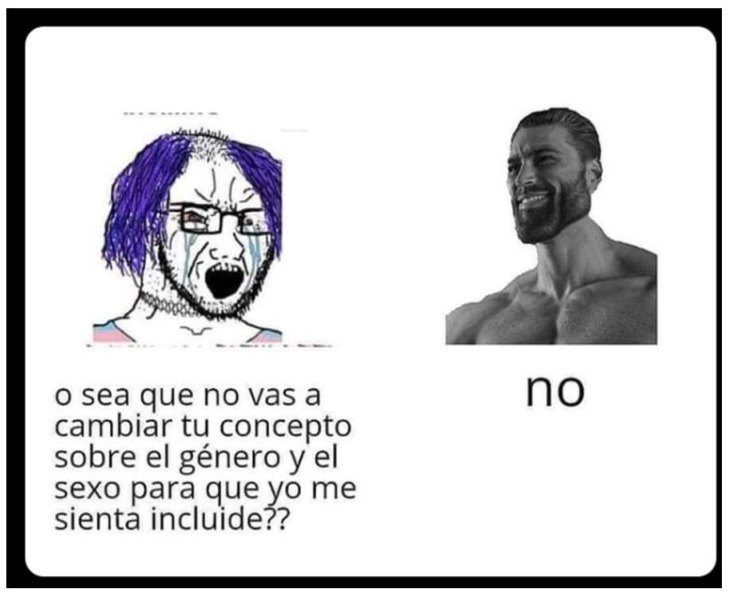
Queer Virgin vs. GigaChad. “So you’re not going to change your concept of gender and sex so that I feel included?”.

An illustrative example (Figure 4) contrasts a Wojak figure (playing the role of Virgin) with dyed hair and emotional expression, demanding recognition through changes to concepts of sex and gender, against the iconic Gigachad who simply replies, “No.” The Virgin/NPC side is verbose and affectively unstable, its speech dense with appeals to inclusion and recognition. By contrast, Chad’s laconic refusal functions as a display of epistemic authority: brevity signals clarity, detachment, and sovereignty. The visual economy here is stark—where the out-group is burdened by textual excess and affective intensity, Chad’s minimal speech and stylized composure anchor the meme’s ideological resolution. This contrast exemplifies how semiotic compression reinforces the communicative hierarchy: the fewer the words, the stronger the authority.

Contextual-discursive affordances extend the meme’s ideological readability beyond its compositional structure. A figure with rainbow-coloured hair and teary eyes may be immediately legible to insiders as a caricature of progressive identity—without need for explicit labels. Similarly, references to the Reconquista, 1492, or crusader iconography evoke nationalist imaginaries, infusing otherwise parodic content with symbolic gravity.

These memes presume high-context interpretive competence. They rely on audiences being conversant not only with meme grammar (e.g., Wojak typologies, NPC templates), but also with broader ideological cues. Terms like “replacement,” “se viene,” or “based” function as dog whistles, indexing affective and ideological affiliation. In this way, memes act as gatekeeping mechanisms, rewarding semiotic fluency and marginalizing or ridiculing interpretive outsiders.

The format thus performs semiotic disciplining: it organizes how bodies, affects, and utterances are made to signify, and in doing so, distributes legitimacy across political subjectivities. Chad embodies brevity, composure, and visual strength; Virgin is marked by excess, instability, and symbolic noise. These contrastive codings do not merely represent difference—they aestheticize hierarchy.

**Figure 5:**
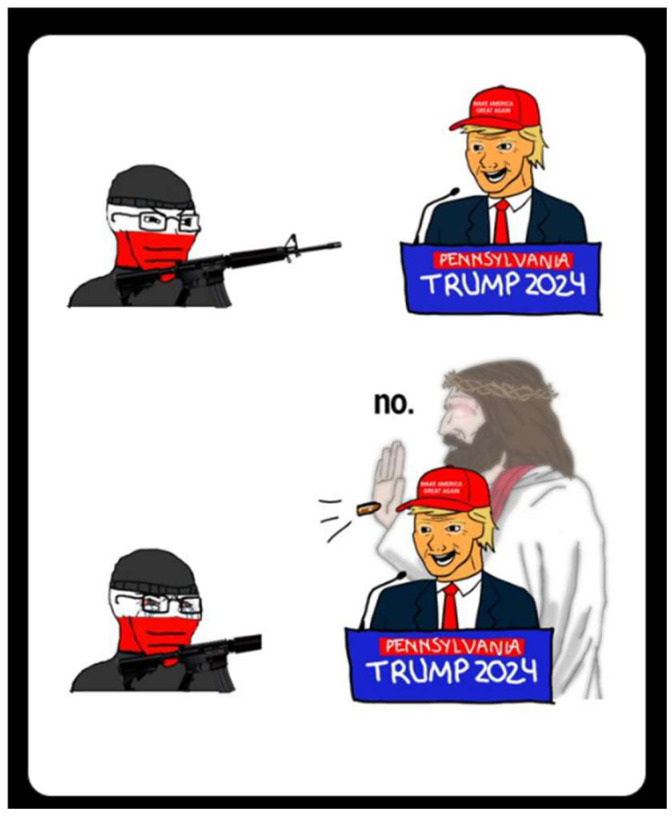
Jesus Chad vs. Shooter Virgin.

This dynamic can also extend beyond Chad’s literal figure, with the archetype mapped onto other characters who embody sovereign strength. In Figure 5, Jesus is represented in Chad-like fashion, deflecting a bullet aimed at Trump with the laconic utterance “no.” Here, Christ’s intervention dramatizes the fusion of nationalist politics with religious authority: Chad’s brevity, composure, and protective force are projected onto a divine archetype. The meme thus exemplifies how the format’s narrative structure can sacralize political struggles, embedding them in a binary of transcendent protection versus conspiratorial threat.

### 3.3. Humour Dynamics Layer (GTVH)

This third analytical layer examines how humour operates within the “Virgin vs. Chad” meme format as a structured communicative strategy according to the GTVH (Attardo, 2024a). The analysis approaches humour as a mechanism that supports alignment, encodes contrastive positions, and shapes affective interpretation. Across the dataset, the GTVH framework helps identify recurring dimensions that contribute to the meme’s ideological function (Table 4):

Script oppositions form the central axis of meaning. Contrasts such as traditionalism versus liberal modernity, or stoicism versus emotional instability, are presented as binary evaluations. These oppositions are not framed as open to negotiation, but as categorical distinctions that demand alignment.

Logical mechanisms involve humour devices such as irony, exaggeration, and reversal. These are used to invert or distort commonly recognized political slogans or cultural expressions in ways that cast the out-group as incoherent, performative, or excessive. The humour often relies on producing a sense of dissonance, resolved through an implicit reaffirmation of the in-group’s perspective.

Situational elements are frequently drawn from ordinary or familiar settings—public transport, sports, social interactions—which are reframed through ideological lenses. These scenarios acquire symbolic weight by referencing broader cultural or historical narratives, which serve to anchor the meme in a more expansive interpretive frame.

Targeting is consistent across the dataset. The memes do not merely point to out-group figures but frame them through stylized depictions that emphasize instability, verbosity, or aesthetic nonconformity. The result is a visual and rhetorical strategy that defines who is outside the bounds of legitimacy.

Narrative strategies typically follow a recognizable rhythm. One figure initiates a claim or action, often framed as misguided or excessive; the other responds, minimally or non-verbally, to reaffirm control or clarity. This structure enables rapid interpretive closure and reinforces the meme’s evaluative stance.

Language use reinforces epistemic asymmetry. Chad’s text is brief, declarative, and visually consistent. Virgin’s is dense, emotionally marked, or parodically stylized. This contrast establishes a communicative hierarchy in which simplicity connotes authenticity, while elaboration is associated with moral or cognitive deviation.

In this context, humour serves to normalize alignment and discredit dissent. It marks acceptable forms of speech and conduct, while ridiculing alternatives as illegitimate or absurd. Rather than introducing ambiguity or playfulness, the humour in these memes supports rigid affective and ideological orientations. It structures how positions are expressed, interpreted, and affiliated with—functioning as a regulatory tool within the meme’s communicative system.

**Figure 6:**
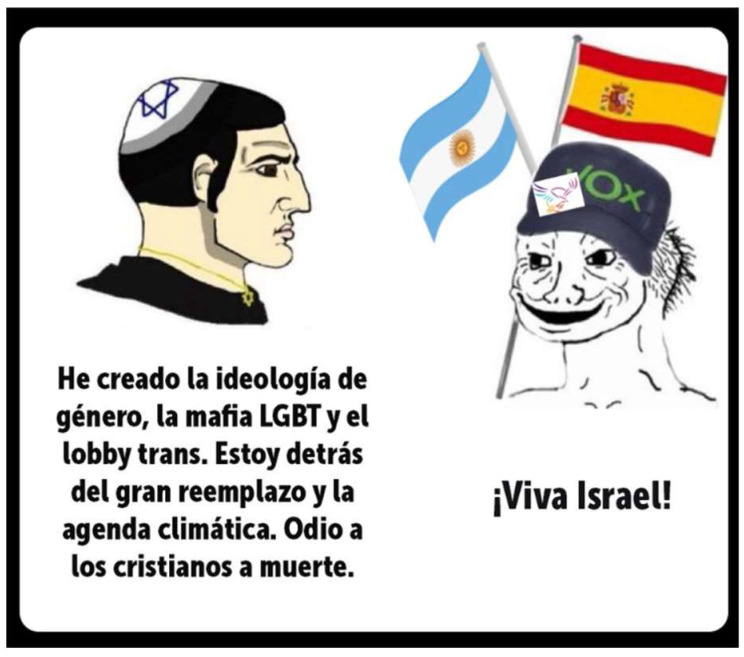
Jew Chad vs. VOX Virgin. A: “I created gender ideology, the LGBT mafia, and the trans lobby. I am behind the great replacement and the climate agenda. I hate Christians to death.” B: “Long live Israel!”.

Figure 6 crystallizes this mechanism is a meme in which a caricatured Jewish figure lists a series of conspiratorial claims—“I created gender ideology, the LGBT mafia, the trans lobby… I am behind the great replacement and the climate agenda. I hate Christians to death”—only for a VOX-supporter Wojak to respond enthusiastically with “¡Viva Israel!” The humour arises from the irony and reversal: the verbose enumeration of antisemitic conspiracy tropes, familiar to far-right discourse, is undercut by the out-group figure’s contradictory alignment with a pro-Israel stance. Within the GTVH framework, the script opposition is between ideological consistency and incoherence; the logical mechanism relies on exposing contradiction through exaggeration and reversal; and the target is less the Jewish caricature than VOX supporters themselves, who are portrayed as naïve or politically servile. The asymmetry of language—extended conspiratorial monologue versus a parodically simplistic slogan—reinforces this evaluative stance. The result is a form of intra-right satire that ridicules VOX for betraying ideological purity, while reaffirming a more radical, antisystemic position.

## 4. Discussion

This study examined how the “Virgin vs. Chad” meme format, within Spanish far-right digital environments, provides a recurring structure for articulating symbolic oppositions between identity positions, behavioural traits, and evaluative stances. By applying a three-layered analytical framework—focused on narrative structure, aesthetic form, and humour mechanisms—the analysis identifies how meaning is organized and rendered interpretable within these meme ecologies. The findings contribute to ongoing debates in media and discourse studies about the role of humour, visual structure, and ideological framing in digital communication (Attardo, 2024a; Fielitz & Ahmed, 2021; Shifman, 2013).

At the narrative level, the meme consistently mobilizes symbolic-relational structures (Glazzard, 2017; Postigo-Fuentes et al., 2024). It stages moralized oppositions between in-group and out-group figures, associates problematizations with the out-group, and affirms in-group values as resolution. These patterns appear as compressed cues rather than developed arguments. In the dataset, Chad consistently functions as a site of coherence—indexed by nationalist, religious, or traditionalist signifiers—while Virgin and related figures are marked through excess, instability, or ideological deviation. This type of narrative framing aligns with prior work on extremist storytelling, which shows how identity constructions rely on simplified moral polarities rather than discursive elaboration (Laclau, 2005; Sun et al., 2025). It also shows the affective logic of these binaries: pride and certainty are attached to Chad, while Virgin and Wojak figures accrue ridicule, shame, or resentment, supporting the view that emotions circulate through figures and help secure group belonging (Ahmed, 2014; Gianolla et al., 2024). These narrative roles are formalized through specific aesthetic choices. 

The second layer of analysis identifies how the meme’s design conventions—dual-panel layout, posture contrast, gaze direction, and speech stylization—enable rapid recognition and interpretive uptake. Such conventions contribute to what Agha (2024) describes as enregisterment, where recurring traits become indexical markers of stance. Their stability is sustained by the processes of viralisation and memeiosis (Attardo, 2024a; Osterroth, 2019). Viralisation reproduces near-identical forms, while memeiosis—repetition with modification—allows for adaptation to new contexts. Across iterations, some features are retained and “frozen,” giving Chad stable associations, while others are reconfigured, enabling semiotisation, or the acquisition of new meanings (Kress & van Leeuwen, 2001). As Zulli and Zulli (2022) argue, ideological readability emerges through contextual uptake, shaped by shared literacies and platform logics. In this case, the Virgin/Chad binary acquires specifically Spanish resonances, mapping global archetypes onto nationalist myths such as the “Verdadero Español” versus “Anti-España” divide (Forti, 2024). This confirms that ideological meaning is stabilized through repetition and circulation, but also reconfigured in response to cultural and historical contexts (De Keulenaar, 2023). These dynamics align with Chang et al.’s (2024) account of generative memesis, in which repetition itself produces social salience, making stylistic patterns recognizable beyond individual authorship.

The third layer examined how mechanisms such as script opposition, incongruity, and reversal operate within this meme format by drawing on shared cultural knowledge and subcultural familiarity (McSwiney & Sengul, 2024; Vandaele, 2010). What makes the meme interpretable as humorous depends on a shared understanding of who is being ridiculed, why their position is marked as incoherent or excessive, and which traits are presented as admirable or laughable. Within these digital settings, humour does not neutralize ideological content—it frames it, modulates its delivery, and marks its recognizability. By presenting antagonisms as recognizable contrasts, humour frames ideological content, modulates its delivery, and ensures its “stickiness” (Ahmed, 2014). This supports research showing that far-right humour acts as both a signal of alignment and a shield against critique, lowering reputational costs while increasing spreadability (Fielitz & Ahmed, 2021; Schmid et al., 2024).

Taken together, the three layers suggest that the “Virgin vs. Chad” format operates as a regularized structure for enacting identity contrast, affective orientation, and symbolic authority. The meme does not simply transmit political ideas but organizes recognition and belonging through repetition, semiotic stabilization, and humour-driven framing. From a discourse-theoretical perspective, memes act as vehicles of power: they fix antagonisms, structure visibility, and distribute legitimacy (Foucault, 1972; Laclau, 2005). As De Keulenaar (2023) and Schmid et al. (2024) have argued, such forms of digital humour can play a central role in how ideological preferences are rendered affectively intuitive and socially sharable—especially in contexts where irony, brevity, and aesthetic recognizability are platform-rewarded.

The Spanish case shows how global meme archetypes acquire new meanings through processes of memeiosis and semiotisation, embedding national myths within transnational templates. In doing so, the analysis supports broader calls for integrating humour studies, multimodal semiotics, and narrative theory in the study of ideological communication online. Memes such as “Virgin vs. Chad” invite interpretation not through argument but through design: they organize attention, prompt identification, and shape relational perception through symbolic structure.

## 5. Conclusions

This article has examined how the “Virgin vs. Chad” meme format functions as a structured resource for the expression of identity, alignment, and relational positioning in Spanish far-right digital contexts. Through a three-layered framework focused on narrative structure, aesthetic form, and humour dynamics, the study has traced how oppositional identities are constructed and evaluated through multimodal stylization.

The findings highlight that the meme’s circulation is sustained through viralisation, memeiosis, and semiotisation. Viralisation reproduces recognizable templates, memeiosis introduces contextual variation, and semiotisation attaches new meanings to stable features. These processes give Chad a relatively fixed indexical value across contexts while allowing for national adaptation, such as its alignment with the “Verdadero Español” against “Anti-España.” In this way, a global format becomes embedded in Spanish extremist narratives, anchoring political antagonisms in historical binaries.

Beyond its ideological content, the meme operates as a form of interpersonal communication. It encodes stance, regulates belonging, and structures group boundaries through humour. The analysis shows that humour in this setting is not incidental but central to how users express affiliation, signal distance, and manage interpretive risk. These functions are consistent with core interpersonal processes such as identity display, boundary management, and affective coordination.

By approaching digital humour as a relational device, the study contributes to bridging humour research with interpersonal communication theory. It supports the view that humour can be analyzed as a patterned form of stance-taking and identity negotiation—particularly in settings where explicit ideological statements are less socially viable. The “Virgin vs. Chad” meme illustrates how humour shapes both how individuals present themselves and how they recognize or reject others.

This framework may be extended to other humour formats to examine how interpersonal meanings are produced and regulated across contexts. In doing so, the article responds to the call for integrating humour with interpersonal communication research, offering a grounded approach to understanding humour not just as content, but as a social and symbolic practice.

Future work could explore how similar meme formats circulate in other national or linguistic contexts, how meme aesthetics evolve across platforms, or how memetic responses across countries challenge dominant narrative structures. Because of the so-called and enregistered “Chad attitude”, it would be interesting to observe whether this framework can be applied to other types of content that are not intentionally humorous. On the other hand, due to the difficulty of regularizing the diffusion of extremist narratives, it could be studied whether the use of this dichotomy (Virgin vs. Chad) could be a sign of diffusion or a signal for action in this type of narratives. Finally, the fact that these memes have a fixed and an acquired meaning could help in the future to automatically detect them.

## Figures and Tables

**Table 1:** Elements of the narrative structure.

Title 1	Title 2
In-group Construction	Chad as crusader, Falangist, VOX voter, “based” nationalist, or ascetic sovereign
Out-group Construction	Feminists, LGBTQ+ individuals, Muslims, immigrants, “soyjaks,” woke NPCs, Jewish caricatures
Problematizations	Cultural erosion, elite betrayal, demographic decline, religious asymmetry
Variations	Gender ideology, trans rights, taxation, climate politics, sports nationalism, bureaucracy
Ideological Perspective	Far-right authoritarianism with elements of libertarianism, identitarianism, or theocracy
Implied Solutions	Patriarchal restoration, national sovereignty, civilizational confrontation, religious reassertion

**Table 2:** Intra-Memetic Affordances.

Aspects	Recurring Patterns
Character Types	Chad = hypermasculine sovereign; Virgin = verbose, feminized, naïve; Villains = devil Wojak, Jewish caricatures
Layout	Split-panel, timeline sequence, or POV framing to spatialize dichotomies
Speech/Text	Chad: aphorisms or minimal speech; Virgin: verbose, emotional, or slogan-driven
Visual Shorthand	Jawline = strength; tears = ridicule or virtue (context-dependent); rainbow hair = progressive alignment
Meme Grammar	Recurrent templates include “Rebuffed Virgin,” “Historical decline → redemption,” “Ironic slogan reversal”

**Table 3:** Contextual-Discursive Affordances.

Required Knowledge	Illustrative Examples
Ideological Literacy	Familiarity with Wojak variants, “se viene” formats, symbolic use of glowing eyes
Meme Literacy	Historical references, real-time political events (e.g., Trump shooting), symbolic inversions
Intertextual Anchors	Stylized parody of feminist, liberal, or leftist discourse
Speech Enregisterment	Affirmation of ideological insiders; mockery or exclusion of the uninitiated
Normative Gatekeeping	Familiarity with Wojak variants, “se viene” formats, symbolic use of glowing eyes

**Table 4:** Recurrent GTVH Dimensions Across the Dataset.

GTVH Dimension	Application in Memes
Script Opposition	Modernity vs. tradition; weakness vs. strength; relativism vs. faith; discourse vs. action
Logical Mechanism	Irony, reversal, incongruity, and cognitive dissonance (e.g., Chad dancing, “Pasamos”)
Situation	Everyday or political contexts reframed as ideological allegories (e.g., voting, transit, sports)
Target	Progressives, feminists, centrists, Muslims, Jews, liberal men, moderate conservatives
Narrative Strategy	Three-phase structure: setup → ideological reversal → affective payoff
Language	Chad: laconic and aphoristic; Virgin/NPCs: verbose, emotional, or slogan-driven

## Data Availability

In accordance with the ethical requirements outlined in the White Book of Ethics of the ARENAS project (https://arenasproject.eu/results/), the data cannot be deposited in a public repository. However, the data are available upon reasonable request for academic purposes by contacting the author at ana.postigo.fuentes@hhu.de. All data were publicly accessible at the time of collection and analysed in accordance with ethical guidelines for research on open digital content. Personally identifiable information was excluded, and the analysis focused exclusively on symbolic content and communicative patterns. Given the ideologically sensitive nature of the material, the interpretive work was conducted with attention to critical reflexivity and discursive sensitivity.
